# Supplementary material for: 17β-Estradiol Promotes Tumorigenicity Through an Autocrine AREG/EGFR Loop in ER-α-Positive Breast Cancer Cells
Source: Cells. 2025 May 12;14(10):703. doi: 10.3390/cells14100703 (PMC12109764; doi:10.3390/cells14100703)
Supplement: Supplementary file 1 [file cells-14-00703-s001.zip › cells-3597338-supplementary.pdf]

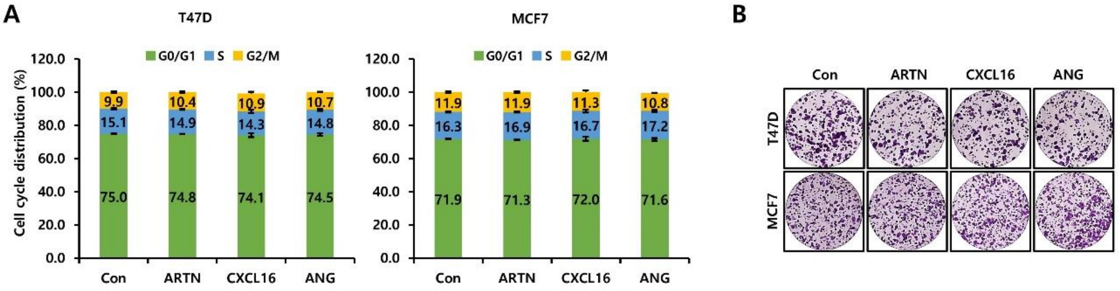

Figure S1. AREG is an essential factor of E2-induced cell proliferation in ER+ breast cancer cells. (A) Each cell type was treated with human recombinant ARTN, CXCL16, and ANG for 24 h. The cell cycle was analyzed by flow cytometry. (B) Cell growth was analyzed using the colony-forming assay. Each cell type was seeded in a six-well plate for 24 h and then treated with 50 ng/mL ARTN, CXCL16, and ANG for 14 days. All experiments were performed in triplicate. All P-values were calculated by unpaired two-tailed Student's t-tests. *P < 0.05, **P < 0.01.

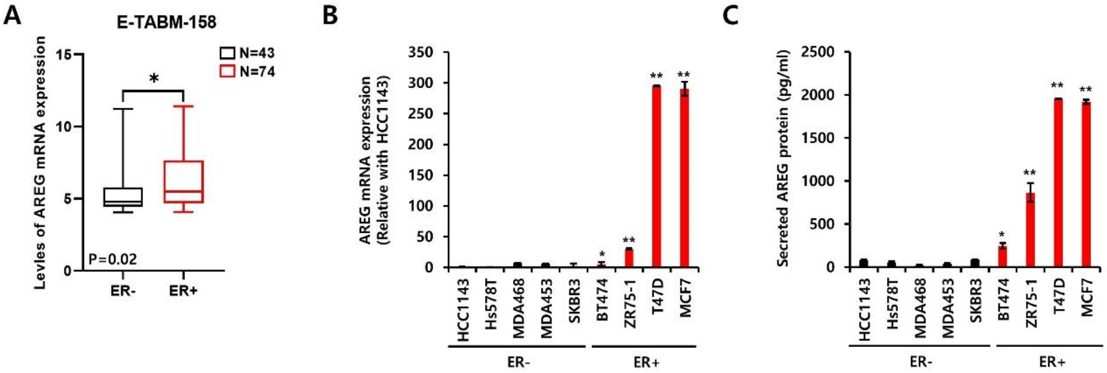

Figure S2. The levels of AREG expression are higher in ER+ breast cancers than in ER- breast cancers. (A) The analysis of E-TABM158 datasets showed that the levels of AREG expression were increased in ER+ breast cancer patients. (B, C) The levels of AREG mRNA and protein expression were analyzed by real-time PCR (B) and ELISA (C) in a variety of breast cancer cells. All experi-ments were performed in triplicate. All P-values were calculated using unpaired two-tailed Stu-dent's t-tests. *P < 0.05, **P < 0.01.

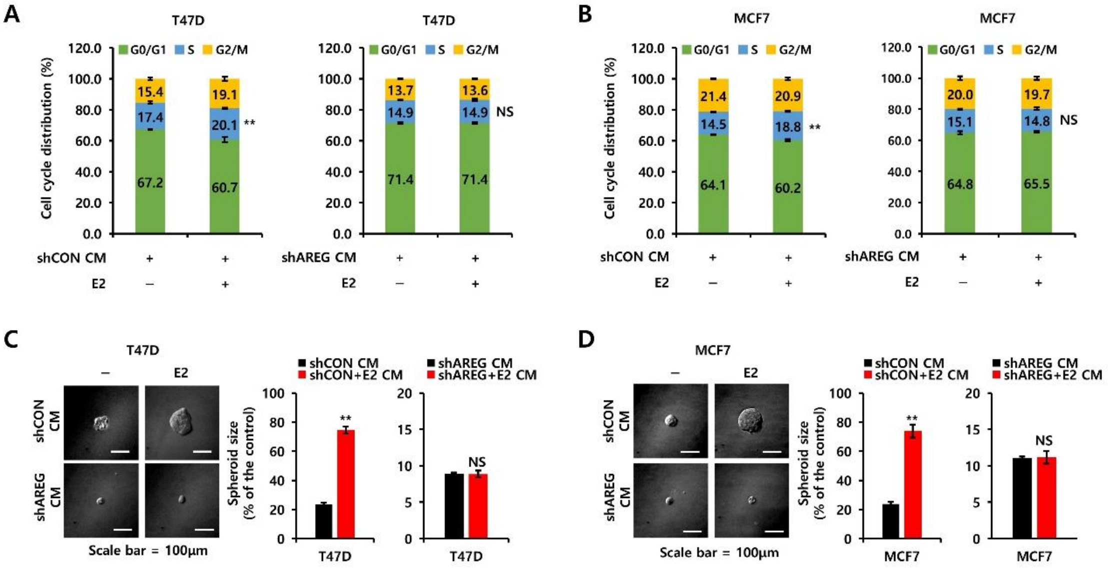

Figure S3. AREG is a potential mediator on the relationship between ER and EGFR. (A, B) Empty vector control or AREG knockdown cells were incubated with or without E2 for 24 hours, after which the conditioned media were collected. Cell cycle analysis was subsequently performed by flow cytometry after 24 hours of conditioned media treatment. (C, D) T47D and MCF7 cells seeded in low-adherent six-well plates in serum-free culture media with growth factors and treated with conditioned media for spheroid formation. Scale bar, 100 μm. All experiments were performed in triplicate. All P-values were calculated using unpaired two-tailed Student’s t-tests. NS : non significant, *P < 0.05, **P < 0.01.
